# Modifiable Barriers to Assessment and Rehabilitation in Justice-Involved Individuals with Self-Reported TBI: The Role of Subjective Sleepiness and Mood

**DOI:** 10.3390/brainsci16050520

**Published:** 2026-05-13

**Authors:** Sarka Turecka Brown, Maddy Pontius, Jennifer Gallagher, Kim A. Gorgens, Gina Signoracci, Marybeth Lehto

**Affiliations:** 1 Graduate School of Professional Psychology, University of Denver, Denver, CO 80210, USA; s.turecka@gmail.com (S.T.B.); maddy.pontius@du.edu (M.P.); jennifer.gallagher@du.edu (J.G.); mblehto@gmail.com (M.L.); 2 Department of Psychiatry, University of Colorado Anschutz, Aurora, CO 80045, USA; gina.signoracci@cuanschutz.edu

**Keywords:** sleepiness, mood, traumatic brain injury, assessment, treatment, criminal justice, cognitive function

## Abstract

**Highlights:**

**What are the main findings?**

- Reported negative mood state (i.e., depression, anxiety, fatigue, restlessness, and anger) was associated with impaired global performance on neurocognitive testing.
- Subjective sleepiness was also related to poorer performance on reaction time tasks.

**What are the implications of the main findings?**

- While sleepiness and mood can contribute to the need for rehabilitation, they may also reduce the likelihood of successful engagement.
- Both sleepiness and mood are modifiable treatment targets, and adapting interventions to accommodate for cognitive inefficiencies can improve overall treatment benefit.

**Abstract:**

**Background/Objectives**: Sleep problems, cognitive deficits, and mood disorders are prevalent in justice-involved populations, especially among individuals with a history of traumatic brain injury (TBI), though the association between these variables remains understudied. This retrospective study examined the relationship between subjective sleepiness and mood state on neuropsychological functioning in a forensic population with self-reported TBI. **Methods**: Data were obtained from 419 inmates and probationers with a self-reported history of TBI using the Ohio State University Traumatic Brain Injury Identification Method and Automated Neuropsychological Assessment Metrics (ANAM). Multiple linear regression models examined associations between cognitive performance across domains (i.e., reaction time, learning, attention, processing speed, working memory, delayed memory, and inhibition) and subjective sleepiness and mood states (i.e., depression, anxiety, fatigue, restlessness, anger, happiness, and vigor) measured by self-report scales embedded in the ANAM. **Results**: Negative mood state was most associated with impaired performance on tests of learning, delayed memory, spatial working memory, and reaction time, as well as global neurocognitive test performance. Subjective sleepiness was predictive of poorer performance on reaction time tasks, while positive mood states were related to better performance on the same task. Regression models were statistically significant (*p* < 0.05), with subjective sleepiness and mood accounting for approximately 1–5% of the variance in cognitive performance. **Conclusions**: Subjective sleepiness and mood symptoms are significantly related to cognitive performance among justice-involved individuals with self-reported TBI. While these factors can contribute to the need for rehabilitation, they may also reduce the likelihood of successful engagement. Importantly, both sleepiness and mood are modifiable treatment targets, and adapting interventions to accommodate cognitive inefficiencies can improve engagement and overall treatment benefit.

## 1. Introduction

Traumatic brain injury (TBI) is defined as a disruption in the normal functioning of the brain caused by an external force such as a bump, blow, or jolt to the head, or penetrating head injury [1]. TBI includes mechanisms such as concussion (i.e., mild, transient disruption in brain function), contusion (i.e., localized bruising of brain tissue), laceration (i.e., tearing of neural structures), or diffuse injury (i.e., widespread shearing of axons due to rotational forces), each of which can result in structural or physiological disturbances that may manifest as diminished or altered states of consciousness [2]. The lifetime prevalence of TBI within the general population has been estimated to be 8.5%, with approximately 2.87 million TBI-related emergency department visits, hospitalizations, and deaths in the United States each year [3,4].

Traumatic brain injury can cause persistent, sometimes life-long, cognitive disturbance, including problems with attention, slowed processing speed, memory impairment, and executive dysfunction [5,6,7,8]. These cognitive deficits are the primary factors contributing to long-term disability [9], highlighting the necessity of accurate evaluation of cognitive function in individuals with TBI.

The results of cognitive assessment can be used to inform treatment recommendations or to justify continued treatment services [10]. However, in justice-involved populations, access to these services is often constrained by systemic barriers including limited resources, stigma surrounding mental health, and disjointed systems [11]. Importantly, neuropsychological variables, such as poor sleep and dysregulated mood, may increase the need for treatment while simultaneously reducing the likelihood of benefitting from standard approaches without accommodations. As a result, cognitive assessment findings carry heightened importance in determining which individuals are identified for and connected with appropriate care and for guiding appropriately tailored interventions. Thus, the stakes for accurate cognitive assessment after brain injury are high, and there are several threats to the validity of that assessment [12].

While there are several fixed characteristics that influence test performance and the validity of data, recent research has focused on more transient or situational factors, including mood and sleep [13,14,15].

Research suggests that mood can adversely affect cognition and pose a threat to the validity of assessment [13,14,15]. Specifically, one study found that participants in negative mood states performed significantly worse on a set of logical reasoning problems than participants who were in an induced positive mood [15]. Another study conducted by Haran et al. [13] found that negative mood states predicted neurocognitive performance during deployment in active-duty service members. Specifically, those authors reported that the increase in negative mood states during deployment may artificially decrease neurocognitive performance.

Sleep is also assumed to influence test performance and affect the validity of test results [16,17]. Sleep deprivation and daytime sleepiness are both related to significantly lower cognitive functioning across multiple cognitive domains including memory, attention, judgment, and decision-making [18]. Van Dongen et al. [19] found that restricting sleep to 4–6 h per night over 14 days in healthy adults led to increased reported sleepiness and impaired attention and working memory compared to 8 h of sleep. Similarly, Haran et al. [13] found that greater self-reported sleepiness among deployed service members was associated with poorer neurocognitive performance across domains of attention, concentration, reaction time, memory, processing speed, and decision-making. Finally, Axelsson et al. [20] demonstrated that experimentally induced sleep restriction (4 h per night across 5 days) resulted in increased sleepiness and impairments in sustained attention, working memory, and executive functioning in healthy adults. Overall, this research suggests that sleepiness has a significant impact on cognitive test results. In these cases, impairments in performance may be mistakenly attributed to cognitive dysfunction, mood, or poor test engagement, when their performance may be instead mediated by sleepiness.

Given this small but robust body of research, it would be logical to assume that special populations known to have poor sleep and a higher prevalence of mood symptoms would be especially vulnerable to poorer cognitive performance, but there is relatively little research in this area. Specifically, forensic populations are especially at-risk for chronic sleep deprivation. According to a 2020 report, there are almost 2.3 million people currently being held in a prison or jail and 3.6 million people on probation in the United States [21]. Research suggests that up to 72% of prisoners exhibit symptoms of insomnia and up to 88% report poor sleep quality [22], compared to 30% of persons in the general population [23]. Furthermore, another study reported that participants recently released from prison had concerns about health problems, lack of access to treatment for sleep disorders, and issues of safety contributing to sleep problems during incarceration and after release [24]. This is presumed to also apply to persons in community corrections systems (e.g., probation).

Forensic populations are also more vulnerable to mood disturbance, such as depression and anxiety. One study found that approximately 91% of inmates reported at least one mental disorder [25]. Specifically, a 2020 literature review reported that major depression has been diagnosed in as many as 37% of inmates globally [26]. Another study reported that inmates in jail have the highest rate of mental illness (60%), compared to inmates in prison [27]. Comparatively, within the general population, the National Institute of Mental Health reported that close to 23% of U.S adults were diagnosed with some form of mental illness in 2021 [28].

Sleepiness and other sleep disturbances are also highly prevalent following TBI, with research indicating that approximately 50% of individuals experience at least one form of sleep disturbance, compared to 41% in the general population [29,30,31]. Individuals with TBI are also more likely to be diagnosed with sleep disorders such as insomnia, which has a 19% increase in likelihood, and sleep apnea, which has a 25% increase in likelihood [31]. In addition to sleep-related issues, mental health problems are also more common after TBI. While approximately 8% of U.S. adults experience a major depressive episode in the general population, about 30% of individuals with TBI develop major depression, often within the first year after injury [32,33,34]. Research also indicates that even mild TBI is associated with an increased risk of developing affective disorders up to four years following injury [35].

The forensic population is also noted to be overrepresented in groups of people at risk for TBI. The prevalence of TBI history in justice-involved populations ranges from 27 to 97% depending on the setting, with an average rate of 54% among individuals on probation, in jail, or involved in problem-solving courts [3,36]. Within community corrections alone (e.g., parole and probation), research has reported the average prevalence of TBI history to be as high as 47% [36]. However, to date, the relationships between sleep disturbance, mental health symptoms, and cognitive performance in this particularly vulnerable group have been understudied. This gap is notable given that many justice-involved individuals are housed in environments that are inherently disruptive to sleep and emotional regulation due to factors such as noise, lighting, and safety concerns [37]. These settings are also primarily designed for security rather than rehabilitation. In contrast, more stable or rehabilitation-oriented settings may be better suited to support recovery, including stabilization of sleep and mood [38]. The limited body of research is flawed by some substantial limitations, such as insufficient control of known confounding factors [13]. Specifically, results may be skewed by the omission of common confounding variables, such as age, gender, education, comorbid diagnoses (i.e., mood, anxiety, or sleep disorders), and history of substance use.

Justice-involved individuals with TBI also face substantial barriers to engaging in and benefiting from rehabilitation services. These include challenges such as short lengths of stay and frequent transitions between incarceration and living in the community, which can disrupt the continuity of care [39]. Access to specialty services such as sleep studies, neurocognitive rehabilitation, and psychological treatment is often limited in under-resourced settings. Individuals must also navigate competing priorities, including securing housing, meeting probation or parole requirements, maintaining employment, and accessing reliable transportation, all of which can interfere with consistent treatment engagement [40]. These challenges are further compounded by stigma surrounding mental health, as well as fragmented coordination across medical, behavioral health, and legal systems [41]. Additionally, sleepiness and mood-related cognitive inefficiencies (e.g., impaired attention, reaction time, learning, decision-making) may further limit justice-involved individuals’ capacity to fully engage in treatment, follow through with recommendations from providers, and benefit from rehabilitative interventions [42,43]. These factors may also reduce individuals’ ability to retain and apply newly learned skills, participate consistently in structured programming, and effectively engage in cognitively demanding interventions, such as psychotherapy or cognitive remediation. Accordingly, these impairments highlight the paradox that individuals with the greatest need for intervention may be the least able to benefit from standard treatment approaches without appropriate accommodations. Within forensic settings, brief cognitive screening is often used to guide treatment planning and rehabilitation recommendations [44]. However, fluctuations in alertness and mood can contribute to variable performance across treatment sessions, complicating clinical assessment, and potentially leading to under-estimation of individuals’ true cognitive and functional capacities. Accordingly, the present study examines whether sleepiness and mood are meaningfully associated with cognitive screening outcomes in a justice-involved population with a self-reported history of TBI.

Given the high prevalence of TBI, sleep disturbance, and mental illness in incarcerated and community corrections populations, the likelihood of sleepiness and mood disturbance is high. As such, there is a related likelihood that sleepiness and mood disturbance serve as neuropsychological assessment variables that may affect cognitive performance and threaten the validity of the assessment, and that inaccurate or invalid assessment can potentially reduce access to and benefit from treatment, despite the high need in this population. The aim of the current study is to address the limitations of past research, and to specifically examine the relationship between self-reported ratings of sleepiness, mood state, and cognitive performance in a population of justice-involved persons with a reported history of TBI, with a particular emphasis on how these factors influence cognitive screening outcomes used to inform accessible, tailored treatment planning and rehabilitation.

## 2. Materials and Methods

### 2.1. Records

A retrospective cross-sectional design was used for this project with data obtained from the TBI Implementation Grant database, DU IRB Protocol # 674894-16. The TBI Implementation Grant database was developed as a research and program development project between the University of Denver, the Colorado Department of Human Services Brain Injury Program, and multiple county jails and probation systems in the Front Range area of Colorado to provide a research database to better understand the needs/vulnerabilities of inmates and probationers with a history of TBI.

Through the Colorado TBI Model [3], inmates and probationers were screened for a TBI history, and if they reported a significant history of TBI, they completed a cognitive screening battery with related recommendations. Study data were collected and managed using the Research Electronic Data Capture (REDCap) electronic data capture tools hosted at the University of Denver. REDCap is a secure, web-based application designed to support data capture for research studies, providing (a) an intuitive interface for validated data entry, (b) audit trails for tracking data manipulation and export procedures, (c) automated export procedures for seamless data downloads to common statistical packages, and (d) procedures for importing data from external sources [45].

Importantly, the incarcerated sample was recruited from county jail settings, where the median length of stay is approximately 14 days. Consequently, individuals frequently transition between custody and community supervision (e.g., probation), resulting in substantial overlap in environmental conditions, access to services, and shared legal and supervision-related stressors across groups. Given this, inmates and probationers were analyzed as a single cohort to reflect the interconnected nature of justice involvement in this population.

#### Participants and Demographic Information of the Database

As part of the TBI Implementation Grant, 4002 adults were screened for reported TBI history and 1818 screened positive. Of those, only 701 persons participated in a computerized neuropsychological screening battery (ANAM), were 18 years of age or older, reported a history of TBI, and consented to allow their responses to be maintained in a deidentified database, meeting inclusion criteria.

**Figure 1:**
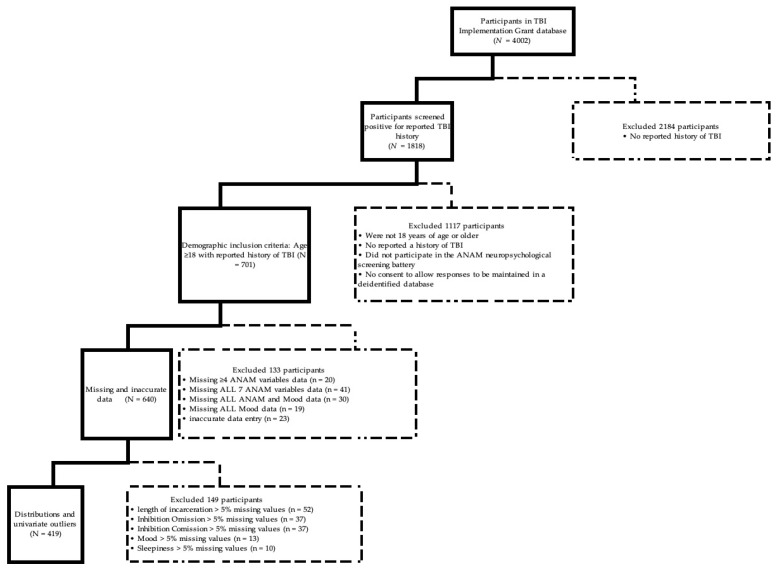
Flowchart of Participant Inclusion and Exclusion. Participants were screened for program eligibility. Participants 18 years or older with a reported history of TBI, based on the Colorado-Revised Ohio State University Traumatic Brain Injury Identification Method, met eligibility criteria for this study. Of these, 419 participated in the ANAM, including completing the Sleepiness Scale and the Mood Scale, and were included in final data analysis.

The final sample of 419 was arrived at by first excluding 133 participants with missing data on all Mood Scale dimensions, at least four of the ANAM subtests, and inaccurate data entry. Next, distributions and univariate outliers were examined, which resulted in exclusion of another 149 participants. No violation of skewness and kurtosis estimates or homoscedasticity was indicated. However, examination of histograms and normality plots indicated minor violations of univariate normality for variables as expected for this population. Outliers were defined as three times the interquartile range above the third quartile or three times below the quartile and were removed prior to analysis. Data imputation was used when more than 5% of total data points were missing, resulting in a final sample size of 419. Information on sample attrition is provided in Figure 1.

Descriptive statistics were calculated for each subtest. Due to normality violations, a sequence of Kendall’s tau-b tests were used to assess differences in subtests, subjective sleepiness, and mood between participant scores. Rank biserial (rsb) correlations were conducted to evaluate effect size and strength of association of results were interpreted using the following criteria: small = 0.10, medium effect = 0.30, and large effect = 0.50 [46].

Prior research examining sleep, mood, and cognitive performance has conducted a priori power analyses using conventional thresholds (e.g., 80% power) and small-to-medium expected effect sizes (e.g., f^2^ ≈ 0.15; refs. [13,47]). Meta-analytic findings further indicate that associations between sleep variables and cognitive outcomes are typically small in magnitude (e.g., g ≈ 0.29; ref. [48]), necessitating larger samples for reliable detection. In the present study, a post hoc observed power analysis was conducted for the multiple regression models using the final analytic sample of 419 participants, three predictors, and α = 0.05. Observed power ranged from 0.74 to 0.99 across the statistically significant models, indicating adequate power to detect the small effects observed in the present analyses within this sample size.

The final sample consists of 147 women and 272 men. Participant demographics, including age, years of education, history of substance use, and self-reported mental health diagnoses, are presented in Table 1. Notably, 250 out of 419 (nearly 60%) individuals in the sample reported a history of an anxiety disorder, while 155 individuals reported a history of a mood disorder (37%) and 12 individuals reported a history of a sleep disorder (approximately 3%).

### 2.2. Assessment Measuresxs

The Ohio State University Traumatic Brain Injury Identification Method is a standardized structured interview that identifies the report of lifetime history of TBI (OSU TBI-ID; refs. [49,50]). It was developed to meet the need for a retrospective, systematic method to identify reported TBI in populations thought to be at risk for TBI and its associated complications and who are unable to complete a full neuropsychological examination or review of records. At the time of this study, there are no widely available neuroimaging or medical diagnostic approaches that are sufficiently sensitive to reliably detect lifetime exposure to repetitive or mild TBI, particularly in cases where injuries were not formally evaluated at the time of occurrence. As a result, structured self-report instruments remain among the most feasible methods for identifying a history of TBI in resource-limited settings where medical documentation is often limited or unavailable, such as jails or probation systems. The OSU TBI-ID has been validated in correctional settings, and Bogner and Corrigan [49] found that the OSU TBI-ID’s test–retest reliability ranges from acceptable to high (≥ 0.60). Its validity is further supported by research showing its results are associated with neuroimaging of brain structure and functioning [50]. Although retrospective self-report methods have limitations, they remain commonly practiced in both research and clinical settings for assessing lifetime history of TBI, particularly in environments with limited access to more comprehensive diagnostic instruments [51,52]. In the Colorado TBI Model, the OSU TBI-ID was modified to reflect only three criteria for significant self-reported TBI history (first, worst, and multiple TBIs). The “Recent Injury” index was removed because most “non-complicated” concussions or mild injuries should resolve within three weeks to three months [53] and the “Other” index was removed because the false positive rate was determined to be too high in pilot studies [51]. The remaining indices—first, worst, and multiple TBIs—are each associated with cognitive deficits in the general population and among a justice-involved population [52]. Accordingly, history of TBI in the current study reflects self-reported, rather than clinically confirmed, injury.

The Automated Neuropsychological Assessment Metrics (Version 4) Core Battery (ANAM; refs. [54,55]) was administered to participants with a significant history of TBI to assess gross cognitive function. It is an automated, computerized neurocognitive test battery that includes a sleepiness scale, mood scales, a questionnaire for self-reporting TBI, and the following subtests: Code Substitution, Matching-to-Sample, Mathematical Processing, Procedural Reaction Time, Simple Reaction Time, Code Substitution Delayed, and Simple Reaction Time Repeated. The ANAM core battery contains a clinically validated embedded performance validity measure [56].

The sleepiness scale is a self-reported measure of sleepiness rated on a 7-point Likert scale, with values closer to 7 indicating increased sleepiness and fatigue. Participants are presented with statements ranging from “Feeling very alert, wide awake, and energetic” to “Very sleepy and cannot stay awake much longer” and are asked to select one statement that best describes their current state. In clinical mental health settings, subjective sleepiness is typically assessed using brief, self-report paper-and-pencil measures due to ease of administration, accessibility, and feasibility in both research and applied healthcare contexts. The Sleepiness Scale is based on one such measure, the Stanford Sleepiness Scale (SSS), created and validated by Hoddes et al. [57] to capture subjective sleepiness state. Importantly, validation research demonstrated that SSS scores were associated with cognitive performance and were sensitive to experimentally induced sleep deprivation, further supporting subjective sleepiness scales’ utility as proxies for functional alertness.

The mood scale is a self-reported measure of seven different mood dimensions: happiness, vigor, restlessness, depression, anxiety, fatigue, and anger. All mood dimensions include six indicators that are rated using a 7-point Likert scale, with higher scores indicating increased endorsement of the respective mood state. This 7-factor model of mood scale has been validated by Johnson et al. [58] as a reliable and valid assessment of mood. Additionally, information on history of mental health diagnoses (i.e., anxiety, mood, and sleep disorders) and symptomology was obtained through unstructured clinical interviews completed by trained graduate clinicians, consistent with standard forensic assessment practices. The interviews included inquiry into past diagnoses, treatment history, and current symptom presentation, with particular attention given to the course, severity, and functional impact of reported symptoms [59]. These data are best conceptualized as provisional, self-reported indicators of psychiatric history.

In clinical contexts, comprehensive interviews, self-report questionnaires, and objective, performance-based measures are considered gold standard approaches for cognitive and mental health assessment, and data collection procedures in the current study were aligned with these practices. Additionally, data quality was strengthened through the application of the Meyers and Volbrecht effort model [60], with all cases demonstrating inconsistent or inadequate effort excluded from analyses. Although self-reported diagnoses are inherently subject to response bias in forensic contexts, these variables were not included as primary predictor variables, but as covariates to account for potential confounding related to baseline psychiatric functioning. This approach allowed subjective mood and sleepiness measures to more directly capture current symptom states in relation to cognitive performance.

### 2.3. Statistical Analyses

All statistical analyses were conducted using the Statistical Package for Social Sciences (SPSS) 25.

#### 2.3.1. Data Reduction

All data were converted to Z-scores. Furthermore, the Z-scores for all subtests (Code Substitution, Matching-to-Sample, Mathematical Processing, Procedural Reaction Time, Simple Reaction Time, Code Substitution Delayed, and Simple Reaction Time Repeated) were averaged to produce a neurocognitive composite score (NCS; ref. [13]). NCS was used in analyses to reduce the risk of Type 1 error, and to minimize floor and ceiling effects [61]. Principle Component Analysis (PCA) was used to address minor mood scale multicollinearity using the loading criterion retention greater than 0.40, a loading difference of 0.10 used for cross-loading elimination. Resultant uncorrelated principal components were used as predictors in regression analyses.

#### 2.3.2. Regression Models

Multiple linear regression models with a simultaneous predictor entry for each ANAM subtest and the NCS were performed using each mood scale principal component and sleepiness as predictors. Regression derived collinearity diagnostics were run to ensure precise estimations, and that reliable neurocognitive performance assessment was due to the relative performance of each predictor. Variable inflation factors (VIFs) for each predictor were used to evaluate multicollinearity. Significant multicollinearity was designated by VIF > 4, with VIFs close to 10 being indicative of serious multicollinearity. No confounding effects were identified for any covariate. Gender, effort, substance abuse history, anxiety, mood, and sleep diagnoses were dichotomized, whereas age and years of education were coded as categorical ranges.

## 3. Results

### 3.1. Descriptive Statistics

Across descriptive analyses, neurocognitive performance was consistently and positively associated with all ANAM subtests, with moderate correlations observed between individual subtests and the neurocognitive composite score on Kendall’s tau-b correlation estimates (τb = 0.45–0.56, *p* < 0.001). In contrast, subjective sleepiness and all individual mood variables demonstrated weak but statistically significant negative associations with cognitive outcomes. Specifically, higher subjective sleepiness and greater endorsement of negative mood states (i.e., anxiety, anger, depression, and fatigue) were associated with lower neurocognitive performance (τb = −0.10 to −0.14, *p* ≤ 0.012), whereas happiness and restlessness showed small positive associations (τb = 0.08, *p* ≤ 0.012).

**Figure 2:**
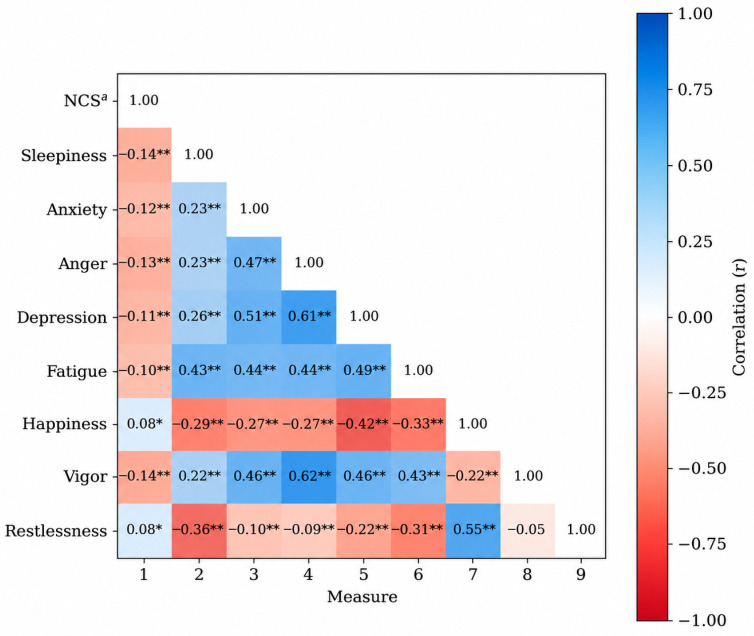
Correlation Between the ANAM Sleepiness, Mood, and Neurocognitive Composite Scores. ^a^ The NCS is a neurocognitive component score calculated by the average of all neurocognitive subtest scores after conversion to Z-scores [13]. The NCS is used in analyses to reduce the risk of Type 1 error and to minimize floor and ceiling effects [61]. * *p* < 0.005 ** *p* < 0.01.

Correlation analyses further clarified these patterns. Subjective sleepiness and most mood variables were negatively associated with neurocognitive performance, including sleepiness (r = −0.14), anxiety (r = −0.12), anger (r = −0.13), depression (r = −0.11), fatigue (r = −0.10), and vigor (r = −0.14). Interestingly, positive correlations were indicated between neurocognitive performance and happiness (r = 0.08) and restlessness (r = 0.08), although restlessness was the only variable that did not demonstrate a statistically significant association (r = −0.05; (see Table 2, Figure 2).

### 3.2. Data Reduction

Kaiser–Meyer–Olkin (KMO) estimates indicated adequate number of cases and significant Bartlett’s chi-square revealed desirable relationships between variables to support PCA (KMO = 0.78; χ^2^ = 1961.71, *p* < 0.001). The PCA converged on a two-factor solution that accounted for 58% and 20% of the total variance respectively. Near equal cross-loading estimates (−0.67, 0.62) for the happiness component prompted a varimax rotation with favorable estimates. As presented in Table 3, Component 1 comprised depression, anxiety, fatigue, restlessness, and anger (negative mood states) while Component 2 included happiness and vigor (positive mood states). These negative and positive mood components together with subjective sleepiness were used as predictors of neurocognitive performance for multiple linear regression analyses.

### 3.3. Regression Models

Across regression analyses, subjective sleepiness and mood variables were significantly associated with neurocognitive performance, although the overall magnitude of these effects was modest. Models predicting the neurocognitive composite score (NCS) and most subtests, including Code Substitution Delayed (CDD), Code Substitution (CDS), Matching to Sample (M2S), Procedural Reaction Time (PRT), Simple Reaction Time (SRT), and Simple Reaction Time Repeated (SRT2), were statistically significant (all *p* < 0.05), whereas the Mathematical Processing (MTH) model was not. Despite statistical significance, subjective sleepiness and mood accounted for a relatively small proportion (~1–5%) of variance across outcomes, with adjusted R^2^ values ranging from 0.02 to 0.05 (NCS = 0.05; CDD = 0.02; CDS = 0.03; M2S = 0.02; PRT = 0.02; SRT = 0.04; SRT2 = 0.04).

Negative mood state was by far the most significant predictor of ANAM throughput scores, demonstrating significant associations with poorer performance on nearly all subtests, with higher endorsement of negative mood corresponding to lower throughput scores. In contrast, SRT2 was the only significant predictor of subjective sleepiness, and SRT was the only significant predictor of positive mood state. No violations of multivariate normality or multicollinearity were indicated based on VIF and tolerance values for subjective sleepiness (VIF = 1.38, tolerance = 0.73), negative mood (VIF = 1.11, tolerance = 0.90), and positive mood (VIF = 1.26, tolerance = 0.79). Overall, while effect sizes were small, the consistency of findings across multiple models suggests that subjective sleepiness and mood are associated with reliable, domain-specific influences on neurocognitive performance in this population (see Table 4).

## 4. Discussion

This study examined the relationship between self-reported sleepiness, mood state, and cognitive performance in a population of justice-involved individuals with a self-reported history of TBI. Importantly, these findings were interpreted in the context of self-reported TBI history, which may differ from clinically confirmed diagnoses, particularly in forensic settings where reporting biases may be present. Clarifying the relationship between these variables is crucial, since both sleep problems and mood disorders are prevalent in a justice-involved population, especially after brain injury, and likely impact effective assessment and treatment planning. For example, prevalence rates of insomnia have a wide range, with some studies reporting 72% of inmates endorsing insomnia symptoms [22]. These same individuals are also much more vulnerable to mood disturbance than the general population. Specifically, up to 37% of persons in the correctional system worldwide have been diagnosed with major depression compared to approximately 23% of the general population [26,28].

TBI is also more prevalent in the justice-involved population, with some studies reporting lifetime prevalence rates as high as 97% depending on the setting [36]. TBI is related to an increased risk for both sleep and mood disturbances, where patients are 19% more likely to be diagnosed with insomnia after TBI and approximately 30% of individuals developed major depression after injury [31,33].

Both sleep and mood disturbance are neuropsychological variables that have been linked to poor cognitive performance, which simultaneously increases the need for intervention and potentially reduces treatment effectiveness in the absence of appropriate accommodations. That deterioration in cognitive performance can belie true cognitive ability and thus contaminate the validity of assessment results. This is particularly consequential given that cognitive screenings are often used to inform treatment planning and justify access to additional resources. Specifically, research suggests that insufficient sleep leads to daytime sleepiness, which is related to significantly impaired performance on cognitive testing [62]. Furthermore, depression and anxiety are also reported to artificially depress scores on neuropsychological testing [63]. These findings suggest that unmeasured sleep and mood disturbances may not only threaten the validity of cognitive screenings, but also contribute to misinformed clinical decision-making. In a forensic context, the stakes for accurate assessments are extremely high, as forensic assessment results often inform important decisions such as competency to stand trial or to make specific treatment recommendations including access to rehabilitation services and accommodations in systems where specialty care is under-resourced.

To date, only one study has quantified the threat of both sleepiness and mood to the validity of assessment [13]. However, that study focused solely on a military population and did not control several potentially confounding factors (i.e., effort, age, and education). The current study focused on another specialty population, justice-involved individuals, and addressed the previous study’s limitations by including a larger sample (n = 419) and controlling for those common confounding variables.

### 4.1. The Effects of Reported Mood State

The results of this study suggest that mood state is most related to neurocognitive functioning. Specifically, negative mood, including depression, anxiety, fatigue, restlessness, and anger, was most associated with impaired performance on tests of learning, delayed memory, spatial working memory, and both trials of a simple reaction time/motor speed task. Overall, negative mood was associated with global neurocognitive impairment.

These data also suggest that, while controlling for negative mood, subjective sleepiness was related to worse performance on a reaction time/motor speed task, whereas positive mood was related to better performance on a different trial of the same task. In this case, while negative mood was associated with impaired performance on both trials of the task, positive mood was associated with higher scores only on the first trial of reaction time/motor speed, and higher reported sleepiness was associated with poorer performance on the second trial of the same subtest. In the ANAM Core Battery, reaction time is assessed over two separate trials. The first trial is the first subtest, and the second trial is the last subtest of the battery.

As previously mentioned, negative mood was related to poorer global neurocognitive test performance, which is consistent with a small but growing body of literature which suggests that a negative mood requires mental effort, which depresses a person’s performance on cognitive testing [64]. Here, the results also found that positive mood was related to better performance on certain cognitive tasks. More specifically, positive mood states were associated with better performance on the first trial of the reaction time task, the first subtest of the battery. It is possible that the experience of happiness and vigor translates into better performance at the start of an assessment battery, but the advantage is transient.

Given the prevalence of depression and anxiety among justice-involved individuals, especially after TBI, it is imperative to screen and identify negative mood symptoms as they may depress test results and threaten the validity or test interpretations and recommendations [13]. In that case, any interpretation of test results could lead to a gross underestimation of true cognitive functioning, including among those with a self-reported history of TBI. This may result in less effective treatment planning or reduced access to appropriate services and accommodations, limiting potential for rehabilitation and treatment benefit. Furthermore, endorsements of happiness and vigor were related to better performance on the first subtest. As such, single test screening may generate an overly optimistic representation of true cognitive ability. In that case, for example, treatment providers may underestimate the level of care needed for treatment.

### 4.2. The Effects of Subjective Sleepiness

The results from the present study also suggest that higher subjective sleepiness ratings, as measured by a single-item self-report measure, were related to poorer performance on reaction time/motor speed tasks specifically; subjective sleepiness was not associated with overall cognitive functioning. This is a departure from conventional assumptions that sleepiness erodes cognitive performance uniformly [65], but it is consistent with a body of research that sleepiness and fatigue have the most prominent effect on reaction time [66].

Additionally, in the present study, subjective sleepiness was related to impaired reaction time/motor speed performance only on the second trial, the last subtest of the battery designed to measure fatigue and endurance. This suggests that reported sleepiness was associated with poorer performance towards the end of a testing sequence. This is consistent with other studies which have found that self-reported sleep problems result in poorer performance on cognitive tasks, but only during the second half of the testing session, likely due to diminished endurance [67].

Although subjective sleepiness and mood accounted for a modest proportion of variance (1–5%), these findings reflect cumulative, clinically actionable influences on cognitive functioning. Associations were observed across multiple domains (i.e., learning, spatial working memory, delayed memory, reaction time), suggesting that subtle, domain-specific inefficiencies can aggregate to produce clinically meaningful disruptions in overall functioning. In forensic contexts, where cognitive data may inform decisions regarding competency to stand trial, treatment access, and rehabilitation, even small performance differences can carry disproportionate downstream consequences. Specifically, there are important implications for treatment planning, as deficits in domains such as attention, learning, and memory may reduce an individual’s ability to acquire, retain, and apply interventions [68]. These same impairments may both increase the need for therapeutic intervention while simultaneously reducing the likelihood that individuals will fully benefit from standard approaches without appropriate accommodations. Identifying impairments in these domains can help support targeted intervention approaches, such as cognitive skills training or cognitive remediation therapies, which aim to improve functioning in areas such as memory and enhance overall treatment responsivity [69]. Overall, examiners should be aware that self-reported sleepiness and mood states may have an effect on test performance during even brief cognitive batteries.

### 4.3. Limitations

Limitations of the present study include the use of self-report (Likert-type scales) of all mood and sleepiness ratings. For sleepiness in particular, the single rating may not accurately reflect true sleepiness or fatigue. Most measures of sleepy states include several questions [70]. For example, the Epworth Sleepiness Scale (ESS) is a reliable and valid assessment of daytime sleepiness that includes eight questions [70,71]. Another measure, the Pittsburgh Sleep Quality Index (PSQI), quantifies sleep disturbance in greater depth. It is an 18-item questionnaire that includes seven component scores: subjective sleep quality, sleep latency, sleep duration, sleep efficiency, sleep disturbances, use of sleep medication, and daytime dysfunction. It is one of the most widely used sleep measures and has been used to assess sleep disturbance after TBI [70].

Another limitation is that the current study included a specialty population of inmates and probationers, but it is possible that, between justice settings, sleepiness and mood are affected in very different ways. For example, individuals on probation may have access to resources that promote better sleep and mood. Conversely, inmates are reported to have poor sleep, but the impact of the duration of incarceration on sleep disturbance has not been studied and was not controlled for in the current study.

Future research with justice-involved populations should control for length of incarceration and should also include a more robust assessment of sleepiness in testing batteries. Also, the replicability of these results should be examined, such as the uniform depression of performance seen in persons with negative mood states. More specifically, research should replicate the differential beneficial impact of positive mood at the start of a battery and the negative effect of subjective sleepiness on attention and processing speed at the end of a battery. Future research should also extend beyond cognitive outcomes to examine how sleepiness and mood influence treatment-related variables, including referral pathways, engagement in rehabilitation services, and functional outcomes following release. Additionally, research is needed to identify and evaluate feasible interventions targeting sleep and mood disturbances to support cognitive functioning and improve rehabilitation outcomes in this population.

### 4.4. Overall Implications

Sleep disruptions and mood disturbances are neuropsychological assessment variables that present a paradox in treatment, particularly among populations that are underserved, such as justice-involved individuals. These factors are associated with increased cognitive impairment, which heightens the need for therapeutic intervention. Yet, the deficits in working memory, learning, delayed memory, and reaction time linked to self-reported poor sleep and mood may impact an individual’s ability to meaningfully engage in, retain, and apply therapeutic interventions. Consequently, those with the greatest need for intervention may be least able to benefit from standardized treatment approaches without the necessary accommodations.

Given these findings, it is important to consider practical, low-resource strategies that may help mitigate subjective sleep and mood-related barriers to cognitive functioning and treatment engagement in justice-related settings. Simple, everyday strategies that accommodate for cognitive deficits can meaningfully improve rehabilitation outcomes, particularly in resource-limited environments. For example, providers can present information in multiple formats (e.g., verbal and written), break large tasks down into smaller steps, and allow additional time for processing and responding. Additionally, frequent verbal reminders, repetition of key information, and brief check-ins with staff can help reinforce memory and organizational skills, while minimizing distractions and simplifying tasks can support attention and comprehension. Incorporating brief grounding or mindfulness exercises and maintaining consistent routines may also help regulate mood and improve engagement [72]. While structural barriers (e.g., environmental noise, safety concerns, lighting) cannot be fully addressed in these settings, targeting more modifiable factors may enhance cognitive functioning and increase individuals’ ability to benefit from rehabilitative interventions.

## 5. Conclusions

Overall, subjective sleepiness and negative mood states are related to poorer performance on cognitive tasks of learning, delayed memory, spatial working memory, and reaction time and motor speed in a forensic population with self-reported TBI. These results add to a very small body of research to suggest that transient or situational assessment variables, particularly negative mood states, may threaten the validity of assessment results, which can limit access to effective rehabilitation services. Importantly, sleepiness and mood differ from some neuropsychological variables in that they are dynamic and modifiable, making even modest associations relevant for treatment planning. Specifically, sleepiness and mood symptoms increase the need for therapeutic intervention, yet the cognitive deficits associated with these factors may limit the ability to successfully engage in and benefit from services without appropriate accommodations. Therefore, small effects on cognitive performance can translate into amplified differences in legal and clinical outcomes, as they operate at the intersection of treatment need and treatment benefit. For these reasons, an evaluation of mood state and subjective ratings of sleepiness should be added to assessment batteries and then taken into consideration when interpreting the results of a neurocognitive assessment, particularly when used to guide treatment and adapt interventions among vulnerable populations. In forensic cases, these variables warrant not only clinical attention, but caution when interpreting and utilizing test results to inform access to rehabilitation and accommodations in treatment.

## Figures and Tables

**Table 1:** Participant Demographics by Gender.

Demographics	Women (*n* = 147)	Men (*n* = 272)	Total (*n =* 419)
	*M* (*SD*)	*M* (*SD*)	*M* (*SD*)
Age	36.06 (9.62)	38.05 (10.97)	37.35 (10.55)
Education (Years)	11.72 (2.17)	12.02 (2.24)	11.92 (2.22)
	*n* (%)	*n* (%)	*n* (%)
Mental Health Diagnosis			155 (37)
Anxiety Disorder	65 (44)	90 (33)	250 (60)
Mood Disorder	105 (71)	145 (53)	12 (3)
Sleep Disorder	5 (3)	7 (3)	155 (37)
History of Substance Use	145 (99)	262 (96)	407 (97)

Note: *n* = number of individuals in the demographic category; % = percentage of the subgroup (women, men, or total) in the demographic category; Mental Health Diagnosis = self-reported.

**Table 2:** Descriptive Statistics and Correlations for Study Variables.

Variable	*M*	*SD*	*r ^b^*	*p*
Neurocognitive Score ^a^	−0.028	0.562	*-*	*-*
Neurocognitive Subtests				
Code Substitution Delayed	84.95	11.20	0.47 **	0.000
Code Substitution	85.35	13.21	0.53 **	0.000
Matching to Sample	87.48	12.73	0.45 **	0.000
Mathematical Processing	83.58	11.47	0.47 **	0.000
Procedural Reaction Time	82.00	17.65	0.57 **	0.000
Simple Reaction Time	77.31	22.35	0.51 **	0.000
Simple Reaction Time Repeated	75.39	22.33	0.56 **	0.000
Sleepiness Scale	2.53	1.302	−0.14 **	0.000
Mood Scale				
Anger	19.43	21.97	−0.12 **	0.000
Anxiety	32.83	23.12	−0.13 **	0.000
Depression	31.56	26.86	−0.11 **	0.001
Fatigue	30.96	23.10	−0.10 **	0.002
Happiness	44.21	24.79	0.08 *	0.011
Vigor	29.65	21.97	−0.14 **	0.000
Restlessness	43.21	21.35	0.08 *	0.012

^a^ The NCS is a neurocognitive composite score calculated as the average of all neurocognitive subtest scores after conversion to Z-scores [13]. The NCS is used in analyses to reduce the risk of Type 1 error and to minimize floor and ceiling effects [61]. ^b^ Significant differences in scores between tests using Kendall’s τ^b^. * *p* < 0.05 ** *p* < 0.01.

**Table 3:** Component Matrix Loadings Extracted by Principal Components Analysis with Varimax Rotation.

Measure	Component Matrix	
	Component 1	Component 2
Depression	**0.91**	−0.12
Anxiety	**0.88**	−0.03
Fatigue	**0.82**	−0.11
Restlessness	**0.80**	−0.37
Anger	**0.69**	−0.44
Happiness	−0.02	**0.95**
Vigor	−0.30	**0.85**

Bold estimates indicate inclusion within corresponding components.

**Table 4:** Multiple Regression Estimates Predicting ANAM Test Performance Summary.

ANAM Subtest ^a^	F-Test	Adjusted R^2^	Sleep	Negative Mood State	Positive Mood State
NCS	*F*_(3, 415)_*=* 7.66, *p* < 0.001 ***	0.05	*t* = −1.95, *p* = 0.052	*t* = −2.99, *p* = 0.003 **	*t* = 1.05, *p* = 0.295
CDD	*F*_(3, 415)_*=* 3.57, *p* = 0.014 *	0.02	*t* = −1.33, *p* = 0.184	*t* = −2.09, *p* = 0.038 *	*t* = −1.84, *p* = 0.066
CDS	*F*_(3, 415)_*=* 4.62, *p* = 0.003 **	0.03	*t* = −1.34, *p* = 0.180	*t* = −2.34, *p* = 0.020 *	*t* = 1.06, *p* = 0.291
M2S	*F*_(3, 415)_*=* 3.84, *p* = 0.010 *	0.02	*t* = −1.37, *p* = 0.173	*t* = −2.16, *p* = 0.031 *	*t* = 0.69, *p* = 0.491
MTH	*F*_(3, 415)_*=* 1.67, *p* = 0.173	0.01	*t* = −1.68, *p* = 0.093	*t* = −0.856, *p* = 0.393	*t* = −0.62, *p* = 0.536
PRT	*F*_(3, 415)_*=* 3.22, *p* = 0.023 *	0.02	*t* = −0.67, *p* = 0.051	*t* = −1.79, *p* = 0.074	*t* = 1.63, *p* = 0.103
SRT	*F*_(3, 415)_*=* 7.42, *p* < 0.001 ***	0.04	*t* = −0.92, *p* = 0.359	*t* = −2.67, *p* = 0.008 **	*t* = 2.62, *p* = 0.009 **
SRT2	*F*_(3, 415)_*=* 7.31, *p* < 0.001 ***	0.04	*t* = −2.08, *p* = 0.038 *	*t* = −2.41, *p* = 0.016 *	*t* = 1.42, *p* = 0.157

^a^ ANAM subtest codes correspond to the following subtest names: NCS (Neurocognitive Score), CDD (Code Substitution Delayed), CDS (Code Substitution), M2S (Matching 2 Sample), MTH (Mathematical Processing), PRT (Procedural Reaction Time), SRT (Simple Reaction Time), SRT2 (Simple Reaction Time Repeated). * *p* < 0.05, ** *p* < 0.01, *** *p* < 0.001.

## Data Availability

No new data were collected in this retrospective study. The protocol, statistical analysis plan, and unidentified participant data from the original study will be available upon request due to privacy reasons.

## References

[1] Traumatic Brain Injury & Concussion—2019. https://www.cdc.gov/traumatic-brain-injury/index.html.

[2] Goldman L., Siddiqui E.M., Khan A., Jahan S., Rehman M.U., Mehan S., Sharma R., Budkin S., Kumar S.N., Sahu A. (2022). Understanding acquired brain injury: A review. Biomedicines.

[3] Gorgens K., Meyer L., Dettmer J. (2020). Data from: Reducing Recidivism for Justice-Involved Individuals with Traumatic Brain Injury.

[4] Traumatic Brain Injury and Concussion—2024. https://www.cdc.gov/traumatic-brain-injury/data-research/index.html.

[5] Lennon M.J., Brooker H., Creese B., Thayanandan T., Rigney G., Aarsland D., Hampshire A., Ballard C., Corbett A., Raymont V. (2023). Lifetime traumatic brain injury and cognitive domain deficits in late life: The protect-TBI cohort study. J. Neurotrauma.

[6] Wilson L., Horton L., Kunzmann K., Sahakian B.J., Newcombe V.F., Stamatakis E.A., Von Steinbuechel N., Cunitz K., Covic A., Maas A. (2021). Understanding the relationship between cognitive performance and function in daily life after traumatic brain injury. J. Neurol. Neurosurg. Psychiatry.

[7] Hacker D., Jones C.A., Yasin E., Preece S., Davies H., Hawkins A., Belli A., Paton E. (2023). Cognitive outcome after complicated mild traumatic brain injury: A literature review and meta-analysis. J. Neurotrauma.

[8] Rabinowitz A.R., Levin H.S. (2014). Cognitive sequelae of traumatic brain injury. Psychiatr. Clin. N. Am..

[9] Vallat-Azouvi C., Swaenepoël M., Ruet A., Bayen E., Ghout I., Nelson G., Pradat-Diehl P., Meaude L., Aegerter P., Charanton J. (2021). Relationships between neuropsychological impairments and functional outcome eight years after severe traumatic brain injury: Results from the Paris-TBI study. Brain Inj..

[10] Lezak M.D., Howieson D.B., Bigler E.D., Tranel D. (2012). Neuropsychological Assessment.

[11] Nishar S., Brumfield E., Mandal S., Vanjani R., Soske J. (2023). “It’s a revolving door”: Understanding the social determinants of mental health as experienced by formerly incarcerated people. Health Justice.

[12] Sherry N., Ernst N., French J.E., Eagle S., Collins M., Kontos A. (2022). Performance validity testing in patients presenting to a specialty clinic with a mild traumatic brain injury. J. Head. Trauma Rehabil..

[13] Haran F., Schumacher P., Markwald R., Handy J., Tsao J. (2019). Relationships between sleepiness, mood, and neurocognitive performance in military personnel. Front. Neurol..

[14] Aita S.L., Hill B.D., Randolph J.J. (2022). Effort is more than suboptimal: Positive aspects of motivation and engagement in neuropsychological assessment. Positive Neuropsychology: Evidence-Based Perspectives on Promoting Brain and Cognitive Health.

[15] Jung N., Wranke C., Hamburger K., Knauff M. (2014). How emotions affect logical reasoning: Evidence from experiments with mood-manipulated participants, spider phobics, and people with exam anxiety. Front. Psychol..

[16] Bernstein J.P.K., DeVito A., Calamia M. (2019). Subjectively and objectively measured sleep predict differing aspects of cognitive functioning in adults. Arch. Clin. Neuropsychol..

[17] Waters F., Bucks R.S. (2011). Neuropsychological effects of sleep loss: Implications for neuropsychologists. J. Int. Neuropsychol. Soc..

[18] Khan M.A., Al-Jahdali H. (2023). The consequences of sleep deprivation on cognitive performance. Neurosci. J..

[19] Van Dongen H., Maislin G., Mullington J., Dinges D. (2003). The cumulative cost of additional wakefulness: Dose-response effects on neurobehavioral functions and sleep physiology from chronic sleep restriction and total sleep deprivation. Sleep.

[20] Axelsson J., Kecklund G., Åkerstedt T., Donofrio P., Lekander M., Ingre M. (2008). Sleepiness and performance in response to repeated sleep restriction and subsequent recovery during semi-laboratory conditions. Chronobiol. Int..

[21] Mass Incarceration: The Whole Pie 2020. https://www.prisonpolicy.org/reports/pie2020.html.

[22] Sheppard N., Hogan L. (2022). Prevalence of insomnia and poor sleep quality in the prison population: A systematic review. J. Sleep Res..

[23] Roth T. (2007). Insomnia: Definition, prevalence, etiology, and consequences. J. Clin. Sleep Med..

[24] Elumn J.E., Li P., Lytell M.S., Garcia M., Wang E.A., Klar Yaggi H. (2024). “What if that’s your last sleep?” a qualitative exploration of the trauma of incarceration and sleep. Sleep Adv..

[25] Favril L., Indig D., Gear C., Wilhelm K. (2020). Mental disorders and risk of suicide attempt in prisoners. Soc. Psychiatry Psychiatr. Epidemiol..

[26] Bedaso A., Ayalew M., Mekonnen N., Duko B. (2020). Global estimates of the prevalence of depression among prisoners: A systematic review and meta-analysis. Depress. Res. Treat..

[27] James D., Glaze L. (2006). Mental Health Problems of Prison and Jail Inmates. Bureau of Justice Statistics Special Reports. https://bjs.ojp.gov/content/pub/pdf/mhppji.pdf.

[28] Mental Illness. https://www.nimh.nih.gov/health/statistics/mental-illness.

[29] Maestri M., Romigi A., Schirru A., Fabbrini M., Gori S., Bonuccelli U., Bonanni E. (2020). Excessive daytime sleepiness and fatigue in neurological disorders. Sleep Breath..

[30] Crichton T., Singh R., Abosi-Appeadu K., Dennis G. (2020). Excessive daytime sleepiness after traumatic brain injury. Brain Inj..

[31] Mathias J., Alvaro P. (2012). Prevalence of sleep disturbances, disorders, and problems following traumatic brain injury: A meta-analysis. Sleep Med..

[32] Major Depression. https://www.nimh.nih.gov/health/statistics/major-depression.

[33] Fakhoury M., Shakkour Z., Kobeissy F., Lawand N. (2021). Depression following traumatic brain injury: A comprehensive overview. Rev. Neurosci..

[34] Hart T., Hoffman J., Pretz C., Kennedy R., Clark A., Brenner L. (2012). A longitudinal study of major and minor depression following traumatic brain injury. Arch. Phys. Med..

[35] Delmonico R.L., Theodore B.R., Sandel M.E., Armstrong M.A., Camicia M. (2022). Prevalence of depression and anxiety disorders following mild traumatic brain injury. PMR.

[36] Gorgens K.A., Meyer L., Dettmer J., Standeven M., Goodwin E., Marchi C., Lyman H. (2021). Traumatic brain injury in community corrections: Prevalence and differences in compliance and long-term outcomes among men and women on probation. Crim. Justice Behav..

[37] Hummel V., Kouka J., Li P., Grimshaw A., Lewis C.-C., Suh E., Paglia G., Michel C., Elumn J.E. (2025). Sleep health behind bars: A global scoping review of sleep in carceral settings. medRxiv.

[38] Camplain R., Hale L., Camplain C., Stageman R., Baldwin J.A. (2022). Changes in sleep quality and housing status among individuals incarcerated in jail. Sleep Health.

[39] Browne C.C., Korobanova D., Chemjong P., Harris A.W.F., Glozier N., Basson J., Spencer S.-J., Dean K. (2022). Continuity of mental health care during the transition from prison to the community following brief periods of imprisonment. Front. Psychiatry.

[40] Dong K.R., Must A., Tang A.M., Beckwith C.G., Stopka T.J. (2018). Competing priorities that rival health in adults on probation in Rhode Island: Substance use recovery, employment, housing, and food intake. BMC Public Health.

[41] Martin K., Taylor A., Howell B., Fox A. (2020). Does criminal justice stigma affect health and health care utilization? A systematic review of public health and medical literature. Int. J. Prison. Health.

[42] Fortier-Brochu É., Beaulieu-Bonneau S., Ivers H., Morin C.M. (2012). Insomnia and daytime cognitive performance: A meta-analysis. Sleep Med. Rev..

[43] Rock P.L., Roiser J.P., Riedel W.J., Blackwell A.D. (2014). Cognitive impairment in depression: A systematic review and meta-analysis. Psychol. Med..

[44] Catalano G., Mason J., Brolan C.E., Loughnan S., Harley D. (2020). Screening prisoners for cognitive impairment—Literature review. J. Intellect. Disabil. Offend. Behav..

[45] Harris P.A., Taylor R., Thielke R., Payne J., Gonzalez N., Conde J.G. (2009). Research electronic data capture (REDCap): A metadata-driven methodology and workflow process for providing translational research informatics support. J. Biomed. Inform..

[46] Cohen J. (1988). Statistical Power Analysis for the Behavioral Sciences.

[47] Siraji M.A., Spitschan M., Kalavally V., Haque S. (2023). Light exposure behaviors predict mood, memory and sleep quality. Sci. Rep..

[48] Crowley R., Alderman E., Javadi A.-H., Tamminen J. (2024). A systematic and meta-analytic review of the impact of sleep restriction on memory formation. Neurosci. Biobehav. Rev..

[49] Corrigan J.D., Bogner J.A. (2007). Initial reliability and validity of the OSU TBI Identification Method. J. Head Trauma Rehabil..

[50] Corrigan J.D., Bogner J.A. (2018). Ohio State University Traumatic Brain Injury Identification Method. Encyclopedia of Clinical Neuropsychology.

[51] Glover N.M., Gorgens K., Meyer L., Dettmer J., Lehto M. (2018). Sensitivity and specificity of the Ohio State University Traumatic Brain Injury Identification Method (OSU-TBI-ID) to neuropsychological impairment. Crim. Justice Behav..

[52] Bogner J.A., Corrigan J.D. (2009). Reliability and validity of the OSU TBI Identification Method with Prisoners. J. Head Trauma Rehabil..

[53] Dikmen S.S., Machamer J.E., Powell J.M., Temkin N.R. (2003). Outcome 3 to 5 years after moderate to severe traumatic brain injury. Arch. Phys. Med. Rehabil..

[54] Vista LifeSciences (2016). Automated Neuropsychological Assessment Metrics (Version 4) Core Battery.

[55] Reeves D., Winter K., Bleiberg J., Kane R. (2007). ANAM genogram: Historical perspectives, description, and current endeavors. Arch. Clin. Neuropsychol..

[56] Roebuck-Spencer T.M., Vincent A.S., Gilliland K., Johnson D.R., Cooper D.B. (2013). Initial clinical validation of an embedded performance validity measure within the Automated Neuropsychological Metrics (ANAM). Arch. Clin. Neuropsychol..

[57] Hoddes E., Zarcone V., Smythe H., Phillips R., Dement W.C. (1973). Quantification of sleepiness: A new approach. Psychophysiology.

[58] Johnson D.R., Vincent A.S., Johnson A.E., Gilliland K., Schlegel R.E. (2008). Reliability and construct validity of the Automated Neuropsychological Assessment Metrics (ANAM) mood scale. Arch. Clin. Neuropsychol..

[59] Gorgens K.A., Kreutzer J., DeLuca J., Caplan B. (2017). Clinical interview. Encyclopedia of Clinical Neuropsychology.

[60] Meyers J.E., Volbrecht M.E. (2003). A validation of multiple malingering detection methods in a large clinical sample. Arch. Clin. Neuropsychol..

[61] Rasmussen L., Larsen K., Houx P., Skovgaard L., Hanning C., Moller J. (2001). The assessment of postoperative cognitive function. Acta Anaesthesiol. Scand..

[62] Wu J., Wu Z., Xie C., Lin Y., Fu Z., Zhu L., Qi W., Wang H. (2023). A high propensity for excessive daytime sleepiness independent of lifestyle is associated with cognitive performance in community-dwelling older adults. Front. Psychiatry.

[63] Keatley E.S., Bombardier C.H., Watson E., Kumar R.G., Novack T., Monden K.R., Dams-O’Conner K. (2023). Cognitive performance, depression, and anxiety one-year after traumatic brain injury. J. Head Trauma Rehabil..

[64] Brose A., Schmiedek F., Lövdén M., Lindenberger U. (2012). Daily variability in working memory is coupled with negative affect: The role of attention and motivation. Emotion.

[65] Lim J., Dinges D. (2010). A meta-analysis of the impact of short-term sleep deprivation on cognitive variables. Psychol. Bull..

[66] García A., Angel J.D., Borrani J., Ramirez C., Valdez P. (2021). Sleep deprivation effects on basic cognitive processes: Which components of attention, working memory, and executive functions are more susceptible to the lack of sleep?. Sleep Sci..

[67] Lehmann P., Eling P., Kastrup A., Grothues O., Hildebrandt H. (2013). Self-reported sleep problems, but not fatigue, lead to decline in sustained attention in MS patients. Mult. Scler..

[68] Trivedi J.K. (2006). Cognitive deficits in psychiatric disorders: Current status. Indian J. Psychiatry.

[69] Taylor R., Cella M., Wykes T. (2025). Cognitive remediation is an evidence-based psychological therapy: Isn’t it time it was treated like one?. Behav. Modif..

[70] Mosti C., Spiers M., Kloss J. (2016). A practical guide to evaluating sleep disturbance in concussion patients. Neurol. Clin. Pract..

[71] Lee J., Chung Y., Waters E., Vedam H. (2020). The Epworth sleepiness scale: Reliably unreliable in a sleep clinic population. J. Sleep Res..

[72] Cognitive Strategies for Community Mental Health. https://cdpsdocs.state.co.us/ccjj/committees/ADTF/Materials/2019-07-10_CCJJ-ADTF_StrategiesGuidebk-CMH_2019-05-06.pdf.

